# Sequence diversity of NanA manifests in distinct enzyme kinetics and inhibitor susceptibility

**DOI:** 10.1038/srep25169

**Published:** 2016-04-29

**Authors:** Zhongli Xu, Susanne von Grafenstein, Elisabeth Walther, Julian E. Fuchs, Klaus R. Liedl, Andreas Sauerbrei, Michaela Schmidtke

**Affiliations:** 1 Jena University Hospital, Department of Virology and Antiviral Therapy, Hans-Knöll-Straße 2, 07745 Jena, Germany; 2 University of Innsbruck, Institute for General, Inorganic and Theoretical Chemistry and Center for Molecular Biosciences Innsbruck (CMBI), Innrain 80/82, 6020 Innsbruck, Austria

## Abstract

*Streptococcus pneumoniae* is the leading pathogen causing bacterial pneumonia and meningitis. Its surface-associated virulence factor neuraminidase A (NanA) promotes the bacterial colonization by removing the terminal sialyl residues from glycoconjugates on eukaryotic cell surface. The predominant role of NanA in the pathogenesis of pneumococci renders it an attractive target for therapeutic intervention. Despite the highly conserved activity of NanA, our alignment of the 11 NanAs revealed the evolutionary diversity of this enzyme. The amino acid substitutions we identified, particularly those in the lectin domain and in the insertion domain next to the catalytic centre triggered our special interest. We synthesised the representative NanAs and the mutagenized derivatives from *E. coli* for enzyme kinetics study and neuraminidase inhibitor susceptibility test. *Via* molecular docking we got a deeper insight into the differences between the two major variants of NanA and their influence on the ligand-target interactions. In addition, our molecular dynamics simulations revealed a prominent intrinsic flexibility of the linker between the active site and the insertion domain, which influences the inhibitor binding. Our findings for the first time associated the primary sequence diversity of NanA with the biochemical properties of the enzyme and with the inhibitory efficiency of neuraminidase inhibitors.

Despite vaccination programs for both older adults and children, *Streptococcus (S.) pneumoniae* remains a substantial cause of morbidity and mortality through a variety of clinical manifestations, such as pneumonia, acute otitis media and sinusitis, severe and potentially life-threatening meningitis and sepsis^1^,^2^. As a leading threat to children under 5 years of age, pneumonia kills more children than any other disease–more than HIV, malaria, and measles combined^3^. The key to pneumococcal disease is the colonisation of the human host^4^,^5^. Numerous surface-associated proteins on streptococci have been shown to interact with eukaryotic cells, extracellular matrix proteins and serum proteins to facilitate this process^5^,^6^. One example of such molecules is the neuraminidase (NA)^5^, which catalyzes the removal of terminal sialic acid residues from various glycoconjugates on the cell surface^7^, or from mucin to decrease the viscosity of the mucus^5^. It also exposes N-acetyl-glycosamine receptors for adherence on the host epithelial cells^8^. In addition, the activity of NA by cleaving glycolipids, glycoproteins, and oligosaccharides provides a carbon source for the bacteria, alters the surface of competing bacteria within the same niche, and/or modifies the function of host clearance glycoproteins^9^. Knockout of NA genes significantly impairs the ability of *S. pneumoniae* (i) to colonize and persist in the nasopharynx and induce otitis media in the Chinchilla Model^8^, (ii) to spread from the nasopharynx to the lungs in mouse^10^, (iii) to survive in nonmucosal sites or cause sepsis^11^.

There are three forms of the pneumococcal NAs, NanA, B and C. All *S. pneumoniae* strains possess NA activity because of a 100% prevalence of NanA anchored on the surface of the bacterium. The *nanB* gene was detected in the majority of studied isolates (96%), and *nanC* was the least prevalent gene (51% in one study)^12^,^13^. The predominance of NanA, corresponding to its essential roles in pathogenesis of pneumococcal strains, renders it an attractive target for structural research^14^,^15^ and therapeutic intervention^7^.

The catalytic domain is the best characterized part of NanA (Fig. 1A). The critical residues, such as the arginine-triad (R347 in the **R**IP motif, R663 and R721)^7^, the nucleophilic tyrosine (Y752) and its associated glutamic acid (E647), and the aspartic acid (D372)^16^,^17^, preserved invariable, as well as the four aspartic boxes^14^.

In addition, the active centre of the NanA is decorated with a so-called insertion domain. It forms a small distinct β-barrel subpocket and sits between the second and third β-strands of the second sheet of the β-propeller fold^10^,^11^. This insertion domain, whose function remains to be clarified, is only found in the three pneumococcal sialidases (NanA, B and C), NanI from *Clostridium (C.) perfringens* and the leech trans-sialidase, with no other homologous structure being found in the protein structure data bank^15^. The catalytic domain of NanA sequence is flanked with an N-terminal lectin domain and a C-terminal membrane binding region (Fig. 1B). The 200 aa-residue lectin domain, also referred to as carbohydrate-binding module (CBM40), specifically recognizes glycans containing terminal α-2,3- or α-2,6-linked sialic acids^16^. A similar domain is also found in NanJ of *C. perfringens*^12^ and the key residues for the sialic acid binding are conserved. Just recently the structure of NanA CBM40 domain was resolved^16^, but the relative orientation of the domain in respect to the catalytic domain is unknown.

Despite the highly conserved enzymatic activity and topology of NanA, the primary sequence revealed its evolutionary diversity, due to its genetic plasticity^17^. *S. pneumoniae* is capable of taking up environmental DNA and incorporating it into its genome through homologous recombination^18^. Interspecies homologous recombination transfer within *nanA* has resulted in high degree of mosaicism. It indicates the importance of this surface protein as a target for host adaptive immune response against the *S. pneumoniae*^17^. Previous studies showed that NanA is susceptible to the influenza virus NA inhibitors (NAIs) oseltamivir and DANA^19^,^20^,^21^,^22^. Oseltamivir and peramivir exert therapeutic effects in influenza virus induced secondary pneumococcal pneumonia in mice^23^,^24^ but the inhibitory potencies of both viral NAIs are much lower against bacterial NanA than viral NA^25^. Moreover, the NanA diversity might lead to distinct susceptibility towards individual NAI. The sequence divergence of NanA therefore not only contributes to the evasion of host defense towards pathogen, but also challenges contemporary medicine for drugs to convey more significant therapeutic effect.

In this study, we investigated the variability of the *nanA* and synthesized five NanAs from three representative strains based on a phylogenetic analysis. We identified their differences in amino acid sequences and for the first time associated them with the biochemical properties of the enzyme, their susceptibility to the NAIs oseltamivir and DANA (a sialic acid derivative), as well as the sensitivity towards bivalent calcium cation (Ca^2+^). In addition, we rationalized the experimental observations with computational modelling and structural investigations, so that our study might guide a more targeted inhibitor screening and shed light on the ligand-target interactions between NAIs and NanA.

## Results

### Primary sequence alignment and phylogenetic analysis of the NanA

After preparing genomic DNA from the *S. pneumoniae* strains, the *nanA* gene was amplified using the primers described previously^26^. We sequenced the PCR products and submitted the deduced amino acid sequences to MEGA 5.2^27^ for phylogenetic analysis (Fig. 2).

The amino acid sequence of D39 NanA (YP_816960)^28^ was acquired from NCBI and included for comparison because its structure was used in docking and dynamic studies. The overall mean distance of the 11 NanAs computed by MEGA equal input model^27^ is 0.051, which is the average proportion of amino acid sites at which the sequences differ (here 5.1%). The phylogenetic tree reconstructed with a Neighbor-Joining method and a bootstrap resampling technique graphically demonstrated major differences in NanA sequences (Fig. 2). The sequence alignment revealed that NanA of CF8919 accommodates a largely substituted N-terminal lectin domain (Fig. 3a). Its catalytic domain was similar to DSM20566 (Fig. 3b) and all other *S. pneumoniae* strains of the largest clade (results not shown). The clade represented by CJ9400 exhibits distinct residue composition in the catalytic domain (Fig. 3b). Its lectin domain was comparable to the other strains studied here. The presence of extra tandem repeats in C-terminal anchor region of NanA formed the clade composed of D39 and DSM14378.

### Sequence variations in the lectin domain of the NanA

The lectin domain or carbohydrate-binding module (CBM40) comprises around 200 residues of the pneumococcal NanA sialidase. It recognizes α-2,3- or α-2,6-linked sialic acids at the glycan terminal^16^, and leads the enzyme to the targeted cell receptor. Alignment of the CBM40 of NanAs from this study with that of the NanJ of *Clostridium perfringens*^12^ revealed conserved key residues for the sialic acid binding (Fig. 3a). They are aforementioned two arginines (red triangle: R), a glutamic acid (blue star: E) and a hydrophobic pocket consisting of phenylalanine, tyrosine/leucine, and tryptophan (green square: F, Y/L, Y and W). Despite the high preservation of residues that tightly interact with the terminal sialic acid among all the NanAs studied, the CBM40 of NanA-CF8919 was found heavily substituted (Fig. 3a). The average pairwise distance between the CBM40s of CF8919 and the rest 10 sequences computed by MEGA equal input model^27^ is 0.223. The alignment of the CBM40 of CF8919 and DSM20566 with a resolved structure of CBM40 (PDB code: 4C1W)^16^ revealed that the sites of residue alterations are mainly solvent exposed (data not shown). Furthermore, a higher fraction of negatively charged amino acids (D and E) and a lower number of positively charged residues (K) were discovered from the CF8919 CBM40. Among the ~40 substituted residues in CBM40 of CF8918, 8 aspartate or glutamate residues *versus* 5 arginine or lysine residues occurred to replace their either oppositely charged or uncharged counterparts.

### Divergent amino acid composition in the catalytic domain of NanA

Phylogenetic alignment of the 11 NanAs clearly divided these isozymes into two clades. An alignment of more than 100 whole NanAs, including samples from UniProt database confirmed this two-clade phylogeny of NanA (data not shown). The smaller clade comprises about ~20% of the sequences and corresponds to the three NanAs CJ9400, CF6857 and PCD8755 in this study. MEGA equal input model^27^ computed the average pairwise distance between the two clades to be 0.144, while the maximum distance within the large clade is only 0.013. Inspecting the alignment of the three representative sequences of CJ9400, CF8919 and DSM20566 (Fig. 3b) revealed that most of the mutations in CJ9400 occurred in the insertion domain. The residues 437–539 are heavily substituted in CJ9400 in comparison to sequences of the larger clade. Structurally, also the other mutation sites are in proximity to the insertion domain: residues 400–420 and residues 550–560 are flanking the subdomain.

Although the β-barrel formed by the insertion domain is topologically isolated, some of the mutations also affect the catalytic site. Here, residues 440–445 form a 3_10_-helix (residues 440–445, Fig. 3b framed region), which was shown to be structurally involved in substrate or inhibitor binding for D39^25^. The residues in the loop, particularly I442 and F443 that contribute to the hydrophobicity, were found in close contact to the acetamido group of sialic acid and derivatives. In this short 6-residue helix, each clade has its typical motif composition, *i.e.* RAV (440–442aa) for the NanA-CJ9400 type and KGI (440–442aa) for the NanA-DSM20566 type. Interestingly, to date experimental structures are only available for proteins bearing the KGI motif.

### Cloning of representative NanAs

**Figure 1:**
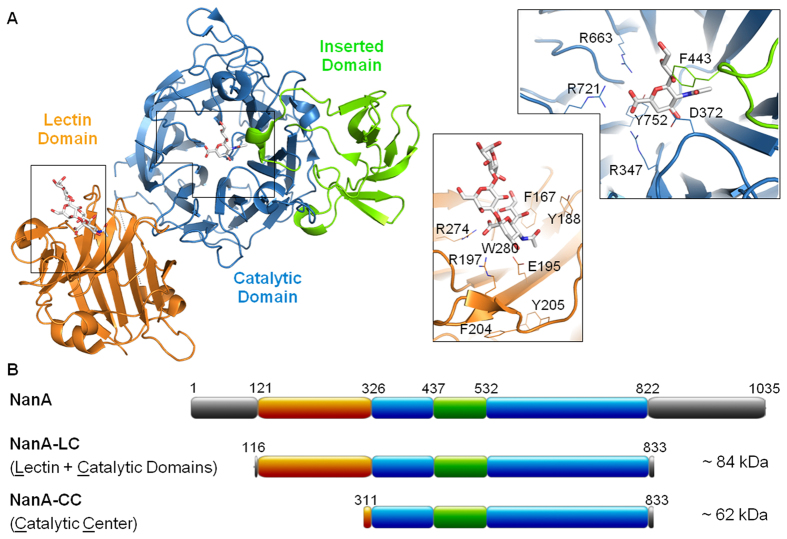
Structure and domain organization of *Streptococcus pneumoniae* NanA. (**A**) Lectin domain (orange) with 2,3-linked sialic acids and the catalytic domain (blue) with a sialic acid derivative (DANA) and the inserted domain (green). Zoom on the binding sites show the conserved active site residues in the catalytic domain and the carbohydrate binding site in the lectin domain. (**B**) Domain organization of the full-length NanA sequence and the NanA-LC and NanA-CC constructs used in this study. ((**A**) generated with PyMOL for X-ray structures with PDB codes 3h73 and 4C1W based on alignment to 2vw0. (**B**) generated with MyDomain – Image Creatore available from http://prosite.expasy.org/mydomains/.)

To further investigate the enzymatic role this primary sequence diversity might play, we selected three representatives, *nanA*s of CF8919, CJ9400 and DSM20566, for cloning and protein expression. A strategy that left out the coding sequence for the N-terminal 115aa non-catalytic residues was employed (Fig. 1B). The synthesized 5′-shortened PCR product encodes the three major domains of NanA, *i.e*. the lectin domain (CBM40), the catalytic domain and the C-terminal cell membrane anchor region. All three *nanA*_116aa variants were successfully cloned into pET-28a and expressed as N-terminal His-tagged recombinant peptide. However, despite many efforts being made to prevent the biodegradation of the ~100 kDa full length NanA, the C-terminal truncated peptide (~80 kDa) was the major product from the heterologous expression in any case (data not shown).

Considering that the C-terminal transmembrane region is not directly involved in the enzyme activity, a construct that encodes only the two functional domains, the lectin (L) and the catalytic (C) domains of NanA was devised. The three constructs are referred to as 8919-LC, 20566-LC, and 9400-LC. A new primer NA_833aaXhoI_rv was used to pair with the primer NA_116aaNdeI_fw to amplify the 2.1 kb fragment from the genome of the three strains. The didomain NanA-LC constructs are ~84 kDa in size and expand from the 116^th^ to the 833^th^ aa of the full length NanA. Furthermore, single-domain NanA-CC constructs (523 aa, ~62 kDa) without lectin functionality and focus on the catalytic centre (CC) only were achieved (Fig. 1B). Because the catalytic domain of NanA-CF8919 is identical to that of NanA-DSM20566, only 20566-CC and 9400-CC were constructed. From both LC and CC clones, polypeptides with expected sizes were expressed as the major products. The N-terminal His-tag installed in each peptide facilitated the purification *via* HisPur Cobalt Spin Column.

### Mutagenesis in the catalytic domain of NanA (20566-CC)

Two primary clades of the catalytic domain of NanA are represented by the more common type 20566-CC and the highly substituted 9400-CC type. As described previously, the differences between these two types are largely embedded in the additional β-barrel subdomain (residues 400–420 and 440–540). To figure out what role the triple mutation of residues 440–442 (KGI versus RAV) plays in the enzyme kinetics and susceptibility towards various NAIs, site-directed mutagenesis was applied to generate three variants of mutant 20566-CCs. The first is the single mutation of the Ile442 residue, which resulted in the construct 20566-CC-KG**V**; the second is the triple substitution product of 20566-CC-**RAV**, and the last is the deletion mutant of 20566-CC-**Δ**KGI.

### Characterization of different constructs of NanA via enzyme kinetics

To investigate the influence of the amino acid substitutions that occurred in different domains of NanA, the five wild-type NanA-LC and NanA-CC constructs and three mutagenized 20566-CCs were subjected to enzyme kinetics studies. Michaelis constant (*K*_m_) was used to characterize each enzyme variant and compare the affinity for fluorescent MUNANA. As listed in Table 1, the *K*_m_ values of each wild-type LC and its correspondent CC construct were very close to each other. An enhancement of affinity for the monovalent MUNANA substrate by the N-terminal lectin domain was not observed. The *K*_m_ value of the 8919-LC is almost equal to that of 20566-LC, however, that of both 9400 constructs are significantly higher than that of their counterparts indicating a reduced affinity for the substrate MUNANA compared to the other two strains.

The mutations generated on the 20566-CC also led to the varying *K*_m_ values. Single mutation I442V in the 3_10_-helix loop region resulted in a slight increase of *K*_m_. Surprisingly, conversion of the entire 20566-type KGI-motif towards 9400-type tri-residue RAV did not lead to an increase of *K*_m_, but drifted the mutant enzyme to a higher affinity for MUNANA than its parent enzyme. The *K*_m_ of 20566-CC-**RAV** is significantly lower than those of 20566-CC-KG**V** and 9400-CC (*p* < 0.050 and *p* < 0.001, respectively; one-way ANOVA, n ≥ 3). In addition, deletion of this three-residue motif considerably reduced the enzyme activity, and the exact *K*_m_ of 20566-CC-**Δ**KGI was immeasurable under the test condition.

### Assessment of the susceptibility of NanA constructs towards further NAIs

Oseltamivir, the most potent competitive inhibitor of NanA known so far^25^,^29^,^30^, was first used to characterize the interaction of the NAIs with different NanA constructs. The binding modality and potency (*K*_i_) was determined *via* steady-state experiments. Oseltamivir mimics and competes with the natural substrate sialic acid and ultimately inactivates the NA function. It is therefore unsurprising that oseltamivir inhibited all NanA constructs (including mutagenized variants; see Table 1) in a specific and competitive manner (SigmaPlot modelling; Fig. S1). The *K*_i_ of oseltamivir towards different NanA constructs mirrored the *K*_m_ values measured previously. Corresponding to the highest *K*_m_ values of the 9400-series, the *K*_i_ values for these enzymes were significantly lower than that of the 8919- or 20566-series (*p* < 0.001; one-way ANOVA, n ≥ 3). The mutagenized variants 20566-CC-KG**V** and 20566-CC-**RAV** with oppositely altered affinity towards MUNANA, exhibited significant difference in the susceptibility to oseltamivir when compared to each other or in comparison to wild type 20566-CC.

To further associate the divergent substitutions in different domains of NanA with the susceptibility to the inhibitor, we used the CL assay to determine the IC_50_ values of each NanA construct. In accordance with the *K*_m_ values characterized above, almost no difference in the IC_50_ values of oseltamivir was observed between NanA-8919 and NanA-20566 constructs (Table 2). Both of the NanA-9400 constructs exhibited significantly lower IC_50_ values (~3-fold). In addition to oseltamivir, the sialic acid based transition state inhibitor DANA^31^ was assessed. A higher concentration of DANA was required to observe the enzyme inhibition than of oseltamivir. Noticeably, in contrast to oseltamivir, the lectin domain of NanA seemed to affect the effectiveness of DANA. To inhibit 50% activity of the LC construct requires more substance than to achieve the same inhibition of the corresponding CC construct. Among the constructs inhibited by DANA, the 8919-LC was most sensitive, while the 9400-constructs exhibited a multiple-fold higher resistance. In spite of the relatively high standard deviation, the reducing power of DANA towards the 9400-variants was in clear contrast to that of oseltamivir.

### Impact of calcium ions on substrate binding affinity of NanA constructs

The effect of calcium ions on each NanA construct was studied *via* enzyme kinetics. The catalytic efficiency of the enzyme was measured in the working MES buffer with or without the addition of 4 mM CaCl_2_. *K*_m_ values of each NanA construct for both conditions are listed in Table 3. Although the enzyme was not inactive in the absence of CaCl_2_, the reaction was indeed less steady, as the *K*_m_ values spread out over a larger range (substantially higher standard deviation) than those under the 4 mM Ca^2+^ condition. Upon the depletion of Ca^2+^, both NanAs of DSM20566 and CJ9400 exhibited ∼2-fold *K*_m_ value rise. Remarkably, the 8919-LC was not sensitive to the Ca^2+^ change.

### Molecular dynamics simulations of NanA identified a flexible region in the active site

We performed molecular dynamics (MD) simulations of NanA to investigate if the active site undergoes conformational transitions which might affect ligand binding. Two systems were investigated: one based on an X-ray structure representing the catalytic domain NanA sequences with the KGI motif, namely D39, the other one based on a model for the catalytic domain of CJ9400 representing those NanAs with RAV motif, Model_CJ9400. Both simulations were performed in presence of the ligand DANA. Comparing the static structures of D39 and Model_CJ9400, we observed that the cavity is about 12 Å^3^ smaller in KGI variants since the larger isoleucine residue (I442) extends in the active site instead of the shorter valine (V442) in the RAV variants (Supplementary Table S1).

The MD simulations indicate that the region around residue 440 is highly flexible (Supplementary Fig. S2 and S3). Flexibility was derived from the MD simulation by two approaches: first, calculating backbone B-factors from the positional fluctuation of backbone atoms in the MD simulation after a single global alignment; second, we performed a local alignment for each residue which allows calculation of a flexibility metric for the protein backbone independent from alignment effects^32^. Both measures confirmed the highly dynamical character of residues 440–450. Interestingly, the simulations share this feature independent from the sequence motif of residue 440–442.

### Modeling molecular interactions between NanA and inhibitors

*In silico* molecular docking was conducted to investigate interaction between the different NanA active site variants and the two NAIs with confirmed binding to the enzyme’s active centre, oseltamivir and DANA. Here we used an induced fit protocol which allows side chain flexibility after initial docking. As the MD simulation indicated a pronounced flexibility in the active site residues 440–442, these residues were additionally allowed adaption of their backbone conformation.

Docking poses for D39 could be compared with X-ray structures (Fig. 4A,B). Quantitatively root mean square deviation (RMSD) of atom positions between the docking pose and X-ray structure indicated successful redocking for D39 (Supplementary Table S2). For DANA the redocking experiment was optimal and the native pose was covered with an RMSD of 0.59 Å (Supplementary Table S2). Oseltamivir carboxylate was redocked with an RMSD of 1.31 Å. A shift of the 3_10_-helix to accommodate the flexible and hydrophobic 3-pentyl group of the ligand was observed. However, the experimental X-ray structure with oseltamivir^25^ showed this rearrangement even more pronounced (Fig. 4B) explaining the higher deviation of the docking pose. The amino group of oseltamivir is positively charged and finds favourable interaction partners in D417. Similarly, oseltamivir can establish this interaction in the homology model of CJ9400 active site (Fig. 4D) and is also reflected by the strongly favourable docking score and estimated free energy of binding for both oseltamivir poses (Supplementary Table S2).

Docking to the Model_CJ9400, especially the data on DANA are striking. In terms of docking score, the best pose obtained did not show the expected orientation of the ligand. In all known NA structures with DANA, the molecule is oriented so that the glycol moiety extends upwards, when the arginine triad is oriented to fix the carboxylate pointing towards the left site (as shown in Fig. 4A). However, for Model_CJ9400 the docking protocol suggests an orientation with the glycol pointing upwards, and a flipped ring plane. Thus, the “best” pose cannot be considered to represent a valid intermediate state of the reaction. Selecting a preferred alternative pose is not unusual, when interpreting docking results. Here, we selected the pose as shown in Fig. 4C, with a classical overall orientation of the DANA molecule. However, the docking result differs to the one for D39, in that the terminal hydroxyl group facing the RAV motif is rearranged and forms less hydrogen bonds. Failure of the standard result and poor scoring value for a reasonable pose, both point towards a worse interaction between DANA and the CJ9400 sequence variant in comparison to D39. Also rescoring with MM/PBSA yields a more favorable prediction for D39 compared to the CJ9400 model (Supplementary Table S3).

## Discussion

The pneumococcal NanA represents a well-characterized virulence factor to colonize the upper and lower respiratory tracts of the host and cause disease^33^,^34^. It was proposed as therapeutic target for antibacterial compounds^7^,^29^,^30^. Due to the natural transformability of pneumococcus, the genetic plasticity of this human pathogen is remarkable^17^,^35^. The significance of the high diversification of *nanA* has been proposed to survive immune surveillance or cause additional inflammation and damage^17^,^36^. For the first time we associate the biochemical properties of the enzyme (e.g. Ca^2+^ dependency and substrate affinity) and its susceptibility to the inhibitors.

**Figure 2:**
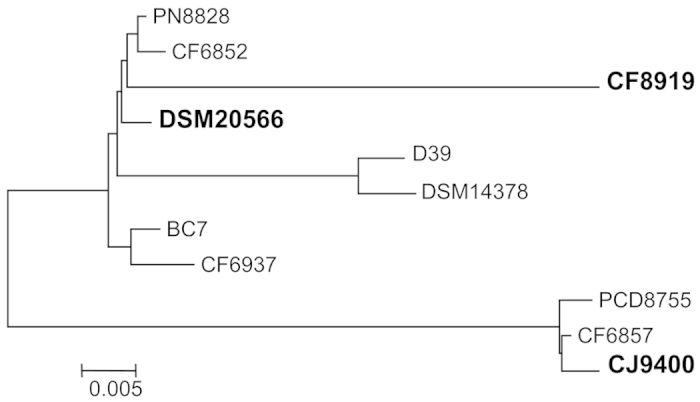
Phylogenetic analysis of the 11 pneumococcal NanAs. A phylogenetic tree was reconstructed based on the alignment of 11 pneumococcal NanAs. Representative sequences which were further investigated are highlighted in bold.

**Figure 3:**
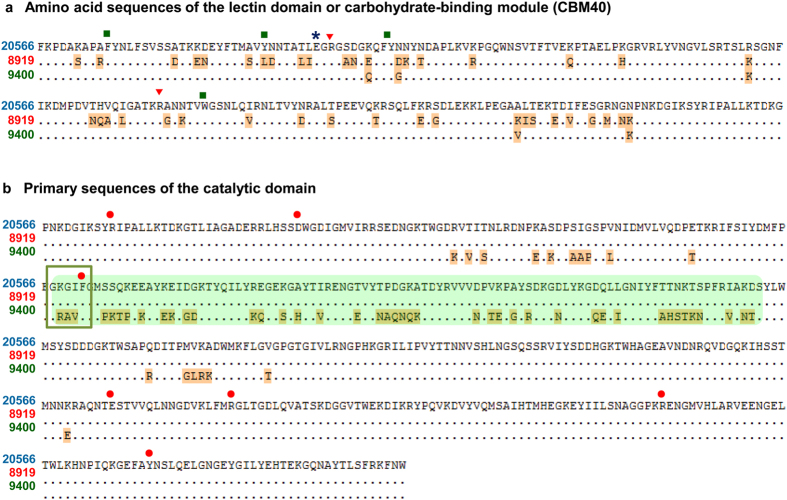
Amino acid alignment of the lectin domain (**a**) and the catalytic domain (**b**) of the three representative NanAs of DSM20566, CF8919 and CJ9400. Residues displaying diversity are labeled with orange background. Amino acids marked with symbols are highly conserved residues for ligand interaction (s. Fig. 1).

**Figure 4:**
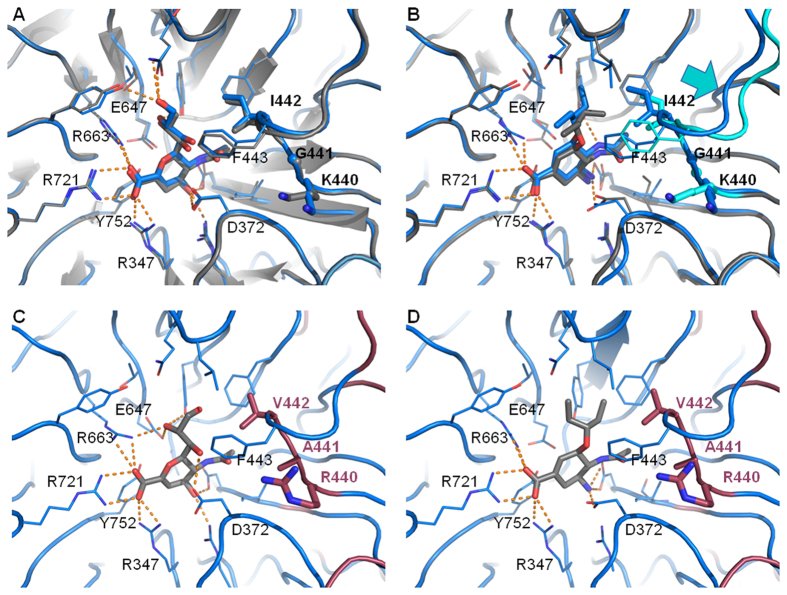
Active site of NanA D39 (**A,B**) and NanA Model_CJ9400 (**C**,**D**) with ligands DANA (**A,C**) and oseltamivir (**B,D**). (**A**) The DANA docking result (blue) for D39 shows high similarity to the experimental pose (grey; PDB code 3h73). (**B**) Docking pose of oseltamivir (blue) compared to the experimental structure (grey; PDB code 2ya8) shows good overlap although the structural shift (arrow) of the flexible region (highlighted in light cyan) is not completely captured in the docking experiment. (**C**) Selected binding pose for DANA in Model_CJ9400 revealed a decrease of hydrogen bonds in comparison to DANA in complex with D39. (**D**) Oseltamivir forms charged interactions with D372 and fits with the pentoxy moiety next to V442 without rearrangement. Hydrogen bonds are highlighted with orange lines. Mutation sites in Model_CJ9400 (**C,D**) are shown in red.

We analysed 11 NanAs in respect to phylogeny and subsequently characterized three sorts of NanAs: i) the DSM20566 constructs representing the majority of NanAs; ii) the CF8919 type harbours highly substituted CBM40 domain, and iii) the CJ9400 variants belong to a smaller clade of NanAs, which differs in the insertion domain adjacent to catalytic centre (Fig. 2).

Calcium was shown to play a critical role for influenza virus NA activity^37^. The NA from *V. cholerae* absolutely requires divalent Ca^2+^ to be active^38^. Ca^2+^ is not a prerequisite for NanA to exert its function. However, increasing calcium concentration (up to 1 mM) did boost the NanA activity by 70%^20^. Similar phenomena were observed with all NanA constructs in this study. Enzyme kinetics showed that the *K*_m_ values of each NanA construct measured under Ca^2+^-null condition spread out over a much larger range than those under the 4 mM Ca^2+^ condition (exhibited as substantially higher standard deviation). This demonstrated that calcium is important in maintaining the stability of the reaction. Furthermore, a decrease in affinity for the substrate was observed for NanA-20566 and NanA-9400 in absence of calcium ions. The reduced *K*_m_ for the substrate MUNANA implies that calcium promotes the binding of the substrate to the catalytic domain. NanA-8919, comprising the highly substituted CBM40, showed no change in *K*_m_ upon lack of CaCl_2_. When the Ca^2+^ concentration is optimal, the *K*_m_ value of the 8919-LC is almost equal to that of 20566-LC, even though the pairwise distance between their CBM40 reaches 0.231. We can therefore conclude that the residue composition of the lectin domain has no direct impact on the enzyme catalytic efficiency. The insensitivity of the CF8919 construct towards the scarceness of calcium ions hints to an involvement of the CBM40 in regulating the NanA calcium sensitivity. The decreased charge of NanA-8919 could have an influence on binding of calcium ions. However, till now there is no report on co-crystallized calcium or other ions in NanA, neither could we identify a calcium binding site when considering the divergent mutations between DSM20566 and CF8919 constructs.

The NanA sequences of CJ9400, PCD8755, and CF6857 share a catalytic domain distinct from the other sequences. The mutations clustered in the insertion domain and the 3_10_-helix next to the binding site. One of the characteristics of this sequence clade is a triple mutation replacing the KGI sequence of residues 440 to 442 by a RAV motif. The motif is also present in 20% of the NanA sequences available on the Uniprot database which confirms the relevance of this NanA sequence clade represented by the NanA-CJ9400 in this study. Remarkably, the *K*_m_ values of both CJ9400 constructs are significantly higher than that of their counterparts, which indicates that NanA-CJ9400 binds the MUNANA substrate less efficiently in comparison to other NanAs. In the docking studies, the structural analogue DANA exhibited less hydrogen bonds with its glycol moiety due to an alternative orientation when faced to the different shape of the sub-pocket in the RAV variant.

To confirm this, the 3-residue motif of 20566-CC was mutated. As expected, a deletion mutation of this section resulted in a loss of function or at least dramatic reduction of activity; probably due to at least partial misfolding in absence of the 3_10_-helix loop. The slightly increased *K*_m_ of mutant 20566-CC-KG**V** indicated that the affinity for MUNANA decreases upon the shortening of branched side chain of the residue. Surprisingly, the *K*_m_ of the triple mutant 20566-CC-**RAV** is significantly lower than those of 20566-CC-KG**V** and 9400-CC (*p* < 0.050 and *p* < 0.001, respectively). The results of MD simulations revealed that the RAV and KGI motifs form part of a highly flexible region undergoing an important rearrangement of the protein backbone. This mobility extends on the insertion domain as seen by comparison of local and global flexibility metrics. We conclude that there is a conformational interplay of the insertion domain and the catalytic site where the KGI or RAV motifs have an important role as flexible linkers. Thus, not only the three-residue motif but also the complete insertion domain appears to modulate the catalytic activity. This role of the insertion domain on the enzyme may explain why the triple mutant of 20566-CC (KGI to RAV) did not result in a switch in to a CJ9400-like kinetic behaviour.

The susceptibility of tested NanAs to oseltamivir and DANA is in good agreement with previous published data^25^,^30^. The determined *K*_i_ and IC_50_ values for oseltamivir are reached in human plasma in pharmacokinetic studies^45^. Structurally, the interaction of oseltamivir is explained with the co-crystal structure of oseltamivir bound to a NanA of D39^25^ and quantitatively reproduced by the favorable docking score in present study. Although the ionic interaction of the amine group is less intensive as in influenza virus NAs, it is expected to be driving the affinity. Interestingly, significantly stronger inhibition by oseltamivir was observed for constructs based on the CJ9400 sequence. As no CJ9400-like structure is available, our docking experiments have to rely on a homology model for structural rationalization of differences between the investigated NanA variants. The exchange of the KGI motif to the RAV motif had the effect, that the shorter isoleucine side chain opens a sub-pocket. The docking pose shows that the Model_CJ9400 active site can easily accommodate the pentoxyl-group of oseltamivir. In consequence the pentoxyl-group may establish hydrophobic interactions over a larger contact surface.

DANA generally is a weaker inhibitor than oseltamivir due to the missing positive feature. As natural transition state analogue of sialic acid, DANA dissociates from the enzyme rapidly upon the flush of substrate, leading to a higher IC_50_ value in the CL assay than oseltamivir. The docking studies and MM/PBSA calculation suggest that DANA shows weaker interaction with the homology model of 9400-CC compared to the 20566-like X-ray structure. However, the mutation data show, that the exchange of KGI in 20566-CC to RAV (20566-CC-**RAV**) did not result in an increased susceptibility towards oseltamivir. Again, this highlights that allosteric effects e.g. mediated by the protein flexibility may drive the distinct enzyme kinetics and drug susceptibility of the variants. The different efficacy of oseltamivir and DANA to different NanA constructs suggests that the naturally occurring sequence variability of NanA might have an important effect on inhibitor susceptibility. We propose that at least one representative of the NanA sequence clade showing the RAV motif accompanied by mutations in the insertion domain should be included when searching for novel bacterial NAIs.

In summary, for the first time we associated the amino acid substitutions in the lectin domain (CBM40) and the inserted domain of NanA with the biochemical properties of the enzyme, which resulted in the differentiated susceptibility towards NAIs as well. In addition, our exploration of the functionally uncharacterized insertion domain *via* site-directed mutagenesis and MD simulations provided us a deeper insight into the influence of this domain on the ligand-target interactions. Especially the region linking the β-barrel of the insertion domain to the active site was shown to impact enzyme-activity. By simulation of protein dynamics, we further identified this region as flexible which complements static experimental structures. This computational modelling and structural investigations may provide guidance for more targeted screening for innovative NAIs and thus, is believed to mediate the consequences of sequence diversity observed for NanA.

## Materials and Methods

### Bacterial strains and cultivation

*S. pneumoniae* strains applied in this work include eight isolates from patients of University Hospital Jena with various symptoms, *i.e.* cystic fibrosis (CF8919, CF6852, CF6857 and CF6937), primary ciliary dyskinesia (PCD8755), pneumonia (PN8828), conjunctivitis (CJ9400) and sepsis (BC7326) described recently^29^; and two strains DSM20566 (ATCC 33400) and DSM14378 (ATCC 6305) from German Collection of Microorganisms and Cell Cultures (DSMZ, Heidelberg). *Escherichia (E.) coli* strains TOP10 and BL21(DE3) were used as host strains for molecular cloning and protein expression of *nanA*, respectively. *S. pneumoniae* was streaked on a Columbia blood agar plate and cultivated overnight at 37 °C in the 5% CO_2_ incubator. *E. coli* strains were inoculated in LB medium overnight at 37 °C and selected by 100 μg/mL of kanamycin.

### NAIs used in this study

Oseltamivir carboxylate GS4071 (oseltamivir; Roche, Switzerland) was dissolved in water as 10 mM stock solutions and stored at −20 °C. The sialic acid analogue 2,3-dehydro-2-deoxy-N-acetylneuraminic acid (DANA) was purchased from Sigma-Aldrich, Deisenhofen, Germany, dissolved in water as 10 mM stock solutions and stored at −20 °C.

### Pneumococcal genomic DNA isolation, sequencing, and cloning of nanA

Pneumococcal genomic DNA was isolated from cells collected from freshly streaked agar plates by using the High Pure PCR Template Preparation Kit (Roche Applied Science) according to the manufacturer’s instructions. Primers NA_116aaNdeI_fw (5′-TGCACGACATATGGAAAATGTC-3′) and NA_Cter_XhoI_rv (5′-TCAAATCTCGAGAATTCTTCTCT-3′) were designed to amplify the coding sequence of NanA (starting from the 116^th^ amino acid) from streptococcal genome. NA_116aaNdeI_fw and NA_311aaNdeI_fw (5′-GTCAACATATGAAACGCTCAG-3′) were respectively paired with NA_833aaXhoI_rv (5′-ATTGAAGGGCTCGAGCCTTG-3′) to generate two NanA variants with shortened C-terminus, namely, NanA-LC (representing **L**ectin and **C**atalytic domains, residue 116–833aa) and NanA-CC (representing **C**atalytic **C**enter, residue 311–833aa) constructs (Fig. 1B). The NdeI/XhoI double-digested PCR product was then ligated to the *E. coli* expression vector pET-28a. Obtained plasmids encoded a series of N-terminal 6× His-tagged NanA peptides.

*nanA* gene fragments were amplified with primers described previously^29^. PCR products were purified with QIAquick PCR Purification Kit (Qiagen,Hilden, Germany) and sequenced by Eurofins (Ebersberg, Germany). All *nanA* clones were completely sequenced (GenBank accession numbers KT893375-82).

### Expression and purification of nanA

A 100 mL culture of *E. coli* BL21(DE3) containing the NanA construct was initially incubated at 37 ^o^C for 3–4 h, then IPTG was added to a final concentration of 0.5 mM for gene expression induction. The broth was further incubated overnight at 25 °C with rigorous shaking and harvested by centrifugation. The resulting cell pellet was subjected to B-PER Protein Extraction Reagents (Thermoscientific) for lysis and protein release. N-terminal His-tagged NanA was purified *via* HisPur Cobalt Spin Column (Thermoscientific) and desalted by Pierce Concentrators PES 30K MWCO. Purified enzyme was preserved in a 40–50% glycerol solution at −20 °C.

### Mutagenesis of NanA-CC

Amino acid substitution was introduced into NanA-CC of the DSM20566 construct *via* respectively designed primer-pair: 20566_KG**V**_fw (5′-CCAGAAGGGAAGGGA**GTC**TTTGGAATGTCTT-3′) and 20566_KG**V**_rv (5′-AAGACATTCCAAA**GAC**TCCCTTCCCTTCTGG-3′) for I442V; 20566_**RAV**_fw (5′-ATGTTCCCAGAAGGG**CGAGCAGTC**TTTGGAATGTCTT-3′) and 20566_**RAV**_rv (5′-AAGACATTCCAAA**GACTGCTCG**CCCTTCTGGGAACAT-3′) for KGI(440-442)RAV; and 20566_KGI_del_fw (5′-ATGTTCCCAGAAGGG**-**TTTGGAATGTCTTCA-3′) and 20566_KGI_del_rv (5′-TGAAGACATTCCAAA**-**CCCTTCTGGGAACAT-3′) for the tri-residue deletion mutation. KAPA HiFi PCR System (KAPA Biosystems) and GeneArt^®^ Site-Directed Mutagenesis System (Invitrogen™) were applied to generate the NanA mutants. Residue substituted NanA20566-CC was then expressed and purified from *E. coli* as described above.

### Enzyme kinetics study using a fluorescence (FL) assay

The enzymatic activity of each NanA construct was measured using the fluorescent 2′-(4-methylumbelliferyl)-a-D-N-acetylneuraminic acid (MUNANA) substrate. The appropriate enzyme dilutions were determined by making 20 μL of serial 2-fold dilutions in a black 96-well microtitre plate and were then assayed in the presence of 30 μL of 100 μM MUNANA (final concentration 60 μM MUNANA) in MES buffer (32.5 mM 2-(N-morpholino)ethanesulfonic acid (MES), 4 mM calcium chloride, pH 6.5). The components of each well were mixed thoroughly and the reaction was terminated by adding 150 μL of stop solution (0.1 M glycine, 25% ethanol, pH 10.7) to each well after 5 min incubation at room temperature. FL signals were detected at an excitation wavelength of 355 nm and emission at 460 nm. Relative fluorescence units (RFU) were converted to the concentration of the product 4-methylumbelliferone (4-MU) according to the 4-MU standard curve. Suitable enzyme dilutions for further testing were determined graphically from the linear section of the enzyme activity curve.

To determine the *K*_m_ values of the recombinant NanA variants, 20 μL of each appropriately diluted (according to the NA activity assay described above) NanA in MES buffer was in triplicate mixed with 30 μL of 50 μM, 100 μM, 200 μM and 400 μM MUNANA, respectively. The converted velocity of the each reaction (μM/min, representing the efficiency of producing 4-MU per minute) and the correspondent substrate concentration were submitted to the Enzyme Kinetics Module of SigmaPlot 12.0 to determine the Michaelis constant (*K*_m_).

To compute the *K*_i_ of the inhibitor, substrate-velocity curves were generated in the presence of several concentrations of inhibitor (including one curve with no inhibitor). Instead of 20 μL of NanA used in the *K*_m_ determination, 10 μL of NanA was premixed with 10 μL of inhibitor before the addition of 30 μL of four concentrations of MUNANA substrate. A competitive inhibition model was fitted to the substrate-velocity data for oseltamivir.

Enzyme kinetics was also used to quantify the influence of Ca^2+^ on NanA activity by measuring the Michaelis constant. Two parallel assay conditions were applied for each NanA-construct, one with 4 mM CaCl_2_ in testing MES buffer, and the other was deprived of any Ca^2+^. The change or the difference of *K*_m_ values was used to interpret the Ca^2+^ effect on the activity of NanA.

### Assessing the inhibitor susceptibility of NanA by a chemiluminescence (CL) assay

The NA-Star^®^ Assay (Applied Biosystems) was used to evaluate the inhibitory activity of oseltamivir and DANA against NanA constructs as recommended by the manufacturer and described recently^30^. IC_50_ values were determined by the curve fitting equation implemented in the CDC JASPR software^46^. Mean and standard deviations of at least three independent assays are shown.

### Structure preparation and homology modeling

The X-ray structure of NanA catalytic domain of the strain D39 (PDB code 3h73, chain B)^39^ was selected to represent the large clade of the NanA sequences with the KGI motif at residues 440–442. Only 5 substitutions between NanA of D39 and DSM20566 occur in the catalytic domain (N337K, E455K, D512N, V606I, and K641R). These variations affect residues distal from the active site. No X-ray structure of NanA is available which could represent the alternative NanA sequences showing a RAV sequence motif in the active site. A homology model of NanA of the CJ9400 strain (Model_CJ9400) was generated based on the X-ray structure of NanA of D39 (PDB code 3h73)^39^. The quality of Model_CJ9400 was ensured by checking the *psi*-*phi* angle plot and close atom contacts. The unusual *cis*-amid configuration of the peptide bond between A776 and Y777 is present in the model as well as in X-ray structures of NanA.

### Molecular dynamics simulations

We investigated the flexibility of the two different NanA structures bound to DANA in the solvated state using all atom molecular dynamics (MD) simulations. Calculations were performed with the simulation package AMBER12^47^ using the force field ff99SBildn^48^ and TIP3P water model^49^. Parameters for the ligand DANA were derived as described earlier for a simulation of the bacterial NA of *Clostridum perfringens*^50^. The simulation was performed on graphical processing units Nvidia GeForce GTX 780 using the pmemd code as implemented in AMBER12^51^. For the current study 100 ns of productive sampling were investigated after employing a rigorous equilibration protocol^52^. Analysis was conducted using AmberTools13^47^ and in-house scripts^32^.

### Molecular docking

*In silico* molecular docking taking into account the flexibility identified by the MD simulations was performed using the induced fit docking tool package of Schrödinger (Induced Fit Docking protocol 2013-3, Glide version 6.1, Prime version 3.4, Schrödinger, LLC, New York, NY, 2013.). The protein structures of D39 and Model_CJ9400 were used as receptor without ligand and crystallographic water molecules, with the definition of the active site center as geometric center of the residues R348, R663, R721, Y752, and F443. The ligands were prepared in MOE (Molecular Operating Environment (MOE), 2012.10; Chemical Computing Group, Inc., Montreal, QC, Canada, 2012) using default criteria for minimization. After ligand pose generation with Glide the induced fit docking protocol optimizes side chain geometries within 5 Å around the ligand. Additionally we selected residues 125–131 to be remodelled during structure refinement. For redocking and optimization, scores were calculated with XP scoring.

To further explain the binding affinity differences between the two strains, MM/PBSA calculation were performed for selected docking poses. The post-processing was performed using a single structure, which was shown to be a practicable and often accurate approach^53^. The complex structures obtained with the induced fit docking protocol were subjected to minimization with AMBER^47^ using a continuum water model. The minimization was performed in three steps, wherein first only hydrogen atoms were minimized while other atoms were harmonically restrained (1000 kcal mol^−1^ Å^−2^), second the restrained on non-hydrogen atoms was reduced to 10 kcal mol^−1^ Å^−2^ and third all atoms were minimized freely. Each step comprised 500 steps of steepest descent and 500 steps of conjugate gradient minimization. The minimized structures were analysed using the Poisson-Boltzman model as implemented in AMBER^47^,^54^.

## Additional Information

**How to cite this article**: Xu, Z. *et al.* Sequence diversity of NanA manifests in distinct enzyme kinetics and inhibitor susceptibility. *Sci. Rep.* **6**, 25169; doi: 10.1038/srep25169 (2016).

## Supplementary Material

Supplementary Information

## Figures and Tables

**Table 1:** *K*_m_ and *K*_i_ values (μM) of each NanA construct indicated the substrate affinity of NanA to MUNANA and its susceptibility to oseltamivir, respectively.

NanA	8919-LC	20566-LC	20566-CC	20566-CC-KGV	20566-CC-RAV	20566-CC-ΔKGI	9400-LC	9400-CC
*K*_m_^a^	24.40 ± 3.85	24.50 ± 2.71	25.97 ± 2.19	32.75 ± 4.78	17.92 ± 1.07	>1000	54.05 ± 9.11	51.27 ± 6.30
*K*_i_^a^	0.33 ± 0.02	0.22 ± 0.03	0.23 ± 0.03	0.12 ± 0.02	0.38 ± 0.04	—b	0.08 ± 0.02	0.07 ± 0.00

^a^Results represent the mean ± s.d. from at least 3 independent determinations for each construct.

^b^Due to the considerable activity reduction of the 20566-CC-ΔKGI, the *K*i was not measured.

**Table 2:** Confirmation of NAI susceptibility of NanA constructs by the chemiluminescence-based NA inhibition assay.

NanA	50% inhibitory concentration (μM)^a^	
	Oseltamivir	DANA
8919-LC	0.34 ± 0.04	1.06 ± 0.44
20566-LC	0.34 ± 0.03	3.80 ± 0.50
20566-CC	0.36 ± 0.05	2.35 ± 0.16
20566-CC-KGV	0.39 ± 0.15	4.56 ± 0.71
20566-CC-RAV	1.27 ± 0.25	0.92 ± 0.71
9400-LC	0.11 ± 0.01	10.06 ± 5.77
9400-CC	0.12 ± 0.01	4.95 ± 2.12

^a^Results represent the mean ± s.d. from at least 3 independent determinations.

**Table 3:** Substrate binding affinity (*K*_m_)^a^ of the NanA constructs under different Ca^2+^ conditions.

Ca^2+^	8919-LC	20566-LC	9400-LC
0	20.41 ± 4.34	45.53 ± 2.05	89.20 ± 2.05
4 mM	25.55 ± 1.89	21.49 ± 0.35^b^	36.18 ± 0.66^b^

^a^Results represent the mean ± s.d. from at least 3 independent determinations.

^b^*K*_m_ values varied when compared with those in Table 1. It might be due to the different batch of preparation of enzyme, substrate or buffer. Important here is to discover the dramatic change of the value of the same construct under different calcium condition.
