# Supplementary material for: Sequence diversity of NanA manifests in distinct enzyme kinetics and inhibitor susceptibility
Source: Sci Rep. 2016 Apr 29;6:25169. doi: 10.1038/srep25169 (PMC4850393; doi:10.1038/srep25169)
Supplement: Supplementary Information [file srep25169-s1.doc]

**Supplementary information**

**Sequence diversity of NanA manifests in distinct enzyme kinetics and inhibitor susceptibility**

Zhongli Xu a, Susanne von Grafenstein b, Elisabeth Walther a, Julian E. Fuchs b, Klaus R. Liedl b, Andreas Sauerbrei a, Michaela Schmidtke a*

a Jena University Hospital, Department of Virology and Antiviral Therapy, Hans-Knöll-Straße 2, 07745 Jena, Germany

b University of Innsbruck, Institute for General, Inorganic and Theoretical Chemistry and Center for Molecular Biosciences Innsbruck (CMBI), Innrain 80/82, 6020 Innsbruck, Austria

*Corresponding author: Schmidtke, Michaela:

Jena University Hospital, Department of Virology and Antiviral Therapy, Hans-Knöll-Straße 2, 07745 Jena, Germany, Tel:+49-3641-9395710, Fax: +49-3641-9395702, E-mail: [michaela.schmidtke@med.uni-jena.de](mailto:michaela.schmidtke@med.uni-jena.de)

20566_CC_KGV

20566_CC_RAV

**Figure S1.** **Lineweaver-Burk plot indicated competitive inhibition of oseltamivir to NanA mutants 20566_CC_KGV and 20566_CC_RAV.** The inhibition assay was performed with oseltamivir concentrations from 0-1 µM.


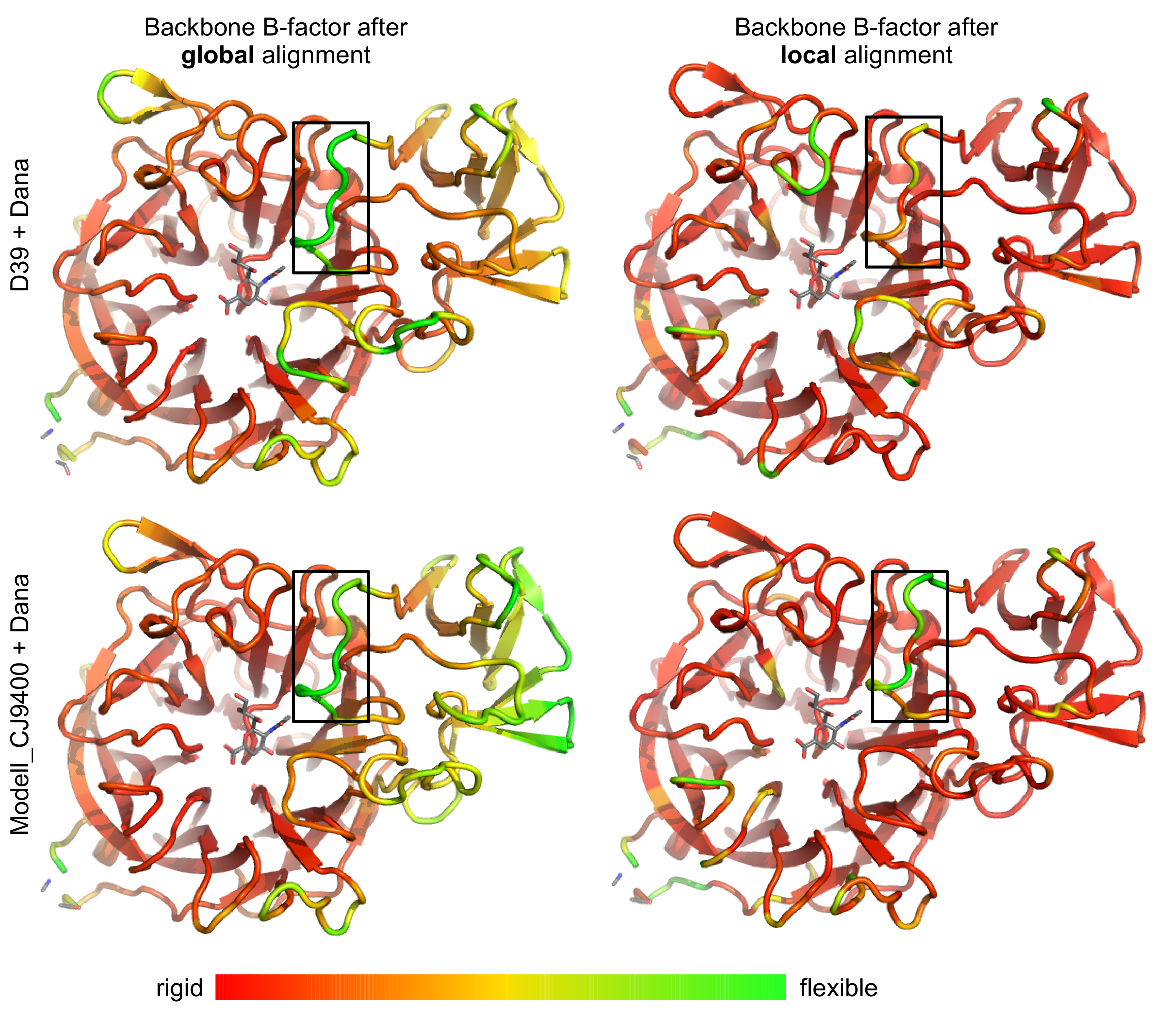


**Supplementary Figure S2. Flexibility of NanA catalytic domains**

Upper part shows results for the simulation of D39 catalytic domain and lower part for the simulation Model_CJ9400.

Left side shows the flexibility pattern from B-factor calculated for the backbone atoms after global alignment. For this flexibility metric the central residues are biased to be more rigid, whereas the outer shell residues are becoming more flexible. Still the squared section of residues 440 to 450 shows a very pronounced flexibility, which according to the visual trajectory analysis is also related to a rearrangement of this loop.

On the right side a local flexibility metrics is displayed, and the B-factor is calculated after the local alignment. Thus, only residues with high intrinsic flexibility are indicated to be flexible. Still, in both systems the residues 440 to 450 show a pronounced relative flexibility and by comparison to the other picture it becomes clear that this region might be a hinge to propagate the flexibility to the insertion domain.

**
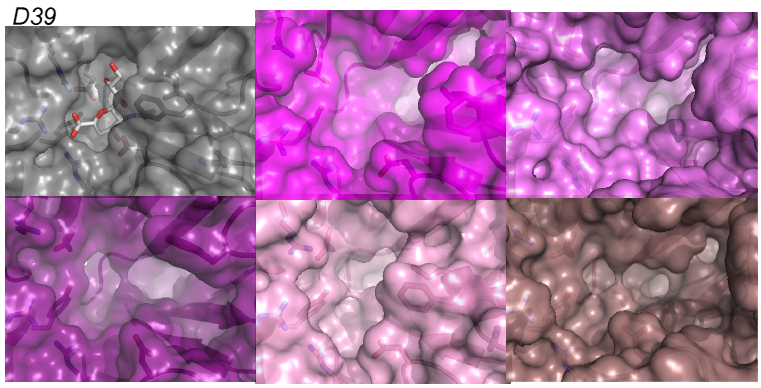
**

***D39***

***Model_CJ9400***

**
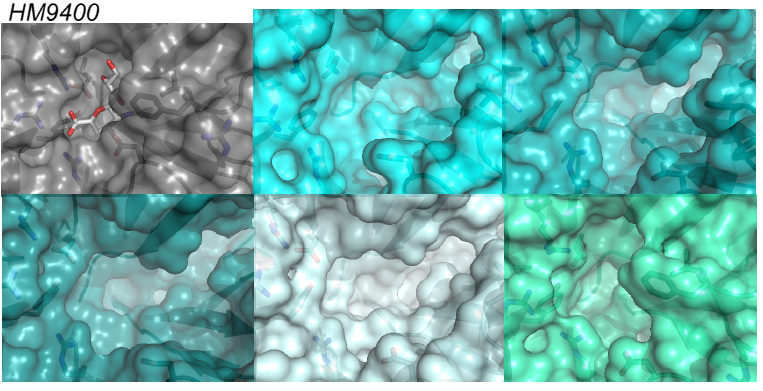
**

**Supplementary Figure S3. Active site cavity for starting structures (grey) and representative cluster conformations from MD simulations of NanA catalytic domain of D39 (upper part) and a homology model for NanA catalytic domain of CJ9400 (lower part).**

The structures from MD simulation show that the active site opens up and especially residues 440-450 explore various conformations.

**Supplementary Table S1.** Calculated cavity volumes.

| Structure | D39 (PDB code 3h73) | Model_CJ9400 |
| --- | --- | --- |
| Pocket volume (Å³) a | 933 | 947 |

a Pocket volumeswere calculated using POVME. The pocket grid was defined by the coordinates of the tyrosine Y752 phenol oxygen as center, a radius of 12 Å and a grid-spacing of 1 Å.
(POVME 2.0: An Enhanced Tool for Determining Pocket Shape and Volume Characteristics; Jacob D. Durrant, Lane Votapka, Jesper Sørensen, and Rommie E. Amaro; *Journal of Chemical Theory and Computation* **2014** *10* (11), 5047-5056 DOI: 10.1021/ct500381c.)

**Supplementary Table S2.** Docking results for induced fit docking of NA inhibitors to NanA structures.

| Structure | D39 | | Model_CJ9400 |
| --- | --- | --- | --- |
| Compound | Ligand RMSDb  (Å) | Docking Score (kcal/mol) | Docking Score  (kcal/mol) |
| DANA besta | 0.628 (3h73) | -9.579 | -9.422 |
| DANA selecteda | 0.592 (3h73) | -9.559 | -8.847 |
| oseltamivir best | 1.310 (2ya8) | -9.976 | -9.888 |

a Besides selection by best scoring value (best), we selected docking poses for similarity to published or expected poses as criteria for pose selection (selected)

b Root mean square deviation of the ligand. Reference structure PDB code is given in brackets for each result.

**Supplementary Table S3.** Results ofMM/PBSA calculation for selected docking results after minimization of the complex structures in comparison with IC­50 values from chemiluminescence assay.

| Structure | D39 | | Model_CJ9400 | |
| --- | --- | --- | --- | --- |
| Compound | ΔG (kcal/mol) | IC­50 (µM) | ΔG (kcal/mol) | IC­50 (µM) |
| DANA selecteda | 56.15a | 2.35 ± 0.16 | 74.68 a | 4.95 ± 2.12 |
| oseltamivir best | -2.52 | 0.36 ± 0.05 | -7.56 | 0.12 ± 0.01 |

a The ranking of the four calculated ΔG values reflects the IC50 values. It should be noted that positive ΔG values are not realistic in view of the experimentally observed inhibition. Quantitative agreement between the experiment and the MM/PBSA re-scoring cannot be expected, as the results may suffer from missing sampling and absence of water molecules bridging protein-ligand interaction.
